# Dual-band and high-efficiency metasurface-based circular-to-linear polarization converter

**DOI:** 10.1371/journal.pone.0286411

**Published:** 2023-06-02

**Authors:** Baoqin Lin, Wenzhun Huang, Jianxin Guo, Yanwen Wang, Rui Zhu, Xiang Ji

**Affiliations:** Xijing University, Xi’an, People’s Republic of China; Universiti Brunei Darussalam, BRUNEI DARUSSALAM

## Abstract

In this paper, to achieve circular-to-linear polarization conversion, a novel polarization converter based on an anisotropic metasurface is proposed. Because the polarization converter is an orthotropic anisotropic structure with a pair of mutually perpendicular symmetric axes *u* and *v*, theoretical analysis shows that the polarization converter can achieve circular-to-linear polarization conversion if its reflection phase difference Δ*φ*_*uv*_ under *u*-polarized and *v*-polarized incidences is close to ±90°. Numerical simulations show that the reflection phase difference Δ*φ*_*uv*_ of the polarization converter is very close to +90° in two separated frequency ranges, so the polarization converter can achieve high-efficiency and dual-band CP-to-LP polarization conversion, it can convert right-handed circular-polarized (RHCP) and left-handed circular-polarized (LHCP) waves into *y*-polarized and *x*-polarized waves respectively in the two separated frequency bands of 8.08–9.27 GHz and 13.80–27.11 GHz; moreover, its polarization conversion rate (PCR) is kept larger than 99.7% in the two frequency bands. Finally, to validate the design, a prototype is manufactured and measured, the measured results are in good agreement with the simulated ones.

## 1. Introduction

The polarization of electromagnetic (EM) wave refers to the characteristics of the direction and amplitude of the electric field in the EM wave changing with time. For various planar EM waves, the spatial trajectory of the endpoint of the time-varying electric field vector at any spatial-point may be a straight line, a circle or an ellipse, so the polarization of EM wave can be divided into the following three basic types: linear polarization (LP), circular polarization (CP) and elliptical polarization (EM). Different polarized EM waves have different propagation characteristics, a suitable polarization should be chosen for EM waves propagating in different propagation environments, so it is of great significance to effectively modulate the polarization of various electromagnetic waves in real time. At present, the LP and CP waves are both widely used, in order to realize the interconversion of LP and CP waves, many LP-to-CP polarization converter based on various metasurfaces have been proposed, these polarization converters can convert LP wave into CP wave in a certain frequency band through reflection or transmission [1–16]; however, the proposed CP-to-LP polarization converter are still very few [17–23], in Ref. [22] and [23], one broadband and one dual-band CP-to-LP polarization converters have been proposed respectively, but the theoretical analysis of the CP-to-LP polarization conversion was still simple.

In this work, a novel CP-to-LP polarization converter is proposed based on an anisotropic metasurface, the polarization converter can achieve high-efficiency and dual-band CP-to-LP polarization conversion under both right-handed circular-polarized (RHCP) and left-handed circular-polarized (LHCP) incidences, the RHCP and LHCP incident waves will be converted to *y*- and *x*-polarized ones respectively in two separated frequency bands. Moreover, for the CP-to-LP polarization conversion, a detailed theoretical analysis is carried out, in which a new type of CP-to-LP reflection coefficients is proposed, the theoretical analysis shows that magnitudes of the CP-to-LP reflection coefficients of the polarization converter can be completely decided by the reflection phase difference Δ*φ*_*uv*_ under *u*-polarized and *v*-polarized incidences; if the reflection phase difference Δ*φ*_*uv*_ can be close to ±90°, the CP-to-LP polarization conversion can be realized.

## 2. Design, analysis and simulation

**Fig 1:**
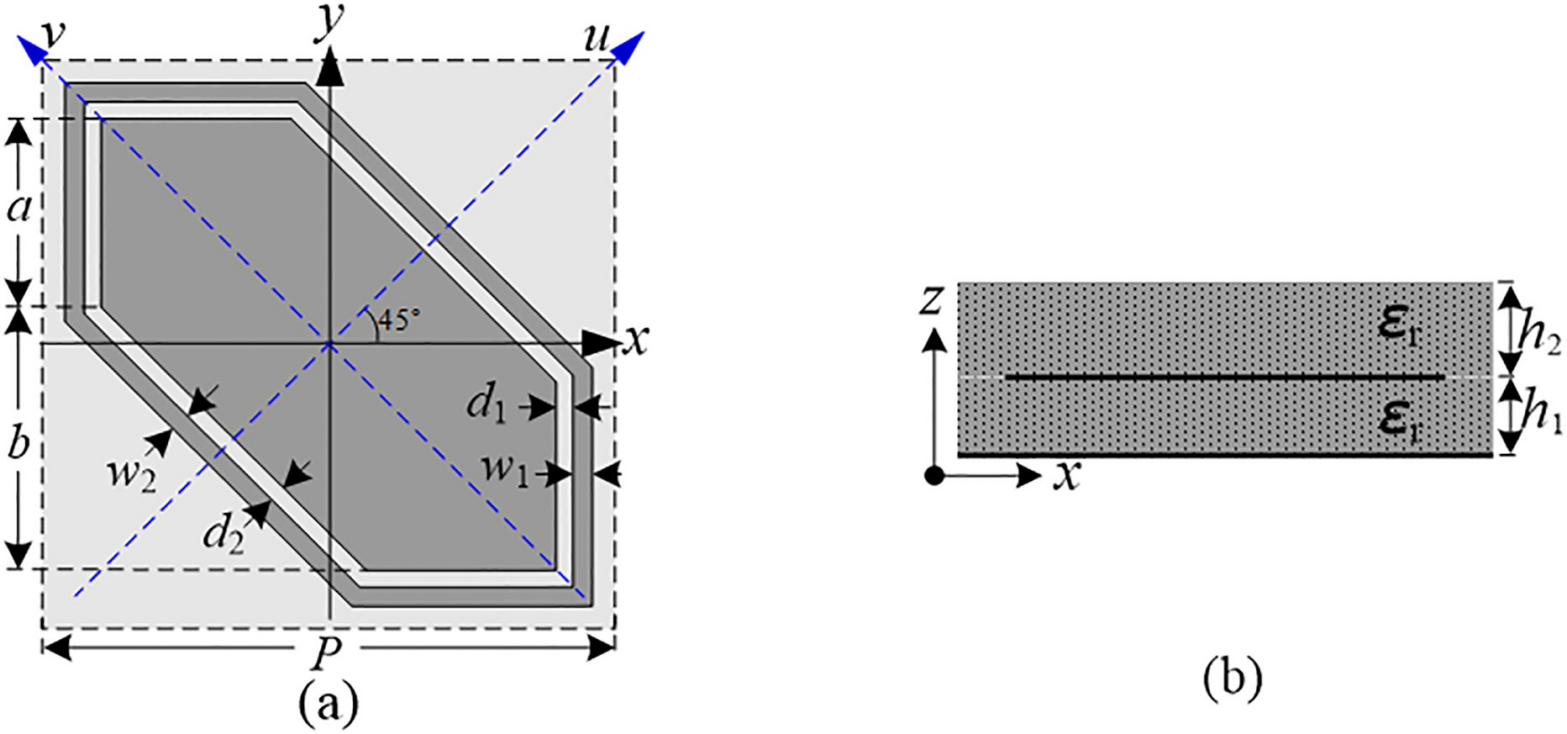
Unit cell of the proposed dual-band CP-to-LP polarization converter. (a) Top view, (b) Side view.

The proposed dual-band CP-to-LP polarization converter is based on a reflective metasurface, which is a two-dimensional periodic structure, one of its unit cells is shown in Fig 1, it is indicated that the polarization converter is an orthotropic anisotropic structure with a pair of symmetric axis *u* and *v* along ±45° directions with respect to the vertical *y* axis. In addition, the geometrical parameters of the unit cell structure are shown in Fig 1, through reasonable selection, these geometrical parameters are chosen as follows: *P* = 8.00 mm, *a* = 2.744 mm, *b* = 2.616 mm, *d*_1_ = 0.25mm, *w*_1_ = 0.20mm, *d*_2_ = 0.20 mm, *w*_2_ = 0.20 mm and *h*_1_ = 2.50mm and *h*_2_ = 2.90 mm; moreover, the two dielectric layers are both chosen as a Polytetrafluoroethylene (PTFE) with a relative permittivity of *ε*_*r*_ = 1.80 and a loss tangent of tan *δ* = 0.0008.

The proposed CP-to-LP polarization converter is symmetrical with respect to both *u* and *v* axes, so no cross-polarized reflection components will exist when it is under the incidence of *u*- and *v*-polarized waves, and its reflection matrix **R**_lin_ in the U-V coordinate system can be expressed as follows:

$${\text{R}}_{\text{lin}}=\left(\begin{array}{cc} r_{uu} & 0\\ 0 & r_{vv} \end{array}\right).$$ (1)

When the incident wave of the CP-to-LP polarization converter is assumed as a RHCP or LHCP one, the incident wave can be expressed as:

$$\begin{array}{ll} E_{RHCP}^{i} & =E_{0}{\hat{e}}_{RHCP}=E_{0}\frac{\sqrt{2}}{2}\left({\hat{e}}_{u}+j{\hat{e}}_{v}\right)\\ E_{LHCP}^{i} & =E_{0}{\hat{e}}_{LHCP}=E_{0}\frac{\sqrt{2}}{2}\left({\hat{e}}_{u}-j{\hat{e}}_{v}\right) \end{array}.$$ (2)

Thus, based on the above reflection matrix **R**_lin_, the total reflected wave under the RHCP and LHCP incidences can be expressed as:

$$\begin{array}{ll} {\left.E_{r}\right|}_{E_{RHCP}^{i}=E_{0}{\hat{e}}_{RHCP}} & =E_{0}\frac{\sqrt{2}}{2}\left(r_{uu}{\hat{e}}_{u}+jr_{vv}{\hat{e}}_{v}\right)=E_{0}\left(\frac{\left(r_{uu}+jr_{vv}\right)}{2}\frac{\sqrt{2}({\hat{e}}_{u}+{\hat{e}}_{v})}{2}+\frac{\left(r_{uu}-jr_{vv}\right)}{2}\frac{\sqrt{2}({\hat{e}}_{u}-{\hat{e}}_{v})}{2}\right)\\ {\left.E_{r}\right|}_{E_{LHCP}^{i}=E_{0}{\hat{e}}_{LHCP}} & =E_{0}\frac{\sqrt{2}}{2}\left(r_{uu}{\hat{e}}_{u}-jr_{vv}{\hat{e}}_{v}\right)=E_{0}\left(\frac{\left(r_{uu}-jr_{vv}\right)}{2}\frac{\sqrt{2}({\hat{e}}_{u}+{\hat{e}}_{v})}{2}+\frac{\left(r_{uu}+jr_{vv}\right)}{2}\frac{\sqrt{2}({\hat{e}}_{u}-{\hat{e}}_{v})}{2}\right) \end{array}.$$ (3)

For the symmetric axes *u* and *v* are along ±45° directions with respect to the *y*-axis, as shown in Fig 1(a), the *y*- and *x*-polarized unit waves can be expressed as ${\hat{e}}_{y}=\frac{\sqrt{2}}{2}({\hat{e}}_{u}+{\hat{e}}_{v})$ and ${\hat{e}}_{x}=\frac{\sqrt{2}}{2}({\hat{e}}_{u}-{\hat{e}}_{v})$, respectively. Now we define two CP-to-LP reflection coefficients as $r_{x-CP}^{}=E_{x}^{r}/E_{CP}^{i}$ and $r_{y-CP}^{}=E_{y}^{r}/E_{CP}^{i}$, Eq (3) shows that the CP-to-LP reflection coefficients under RHCP and LHCP incidences can be determined by the following equation:

$$\begin{array}{ll} r_{y-RHCP} & =r_{x-LHCP}=\left(r_{uu}+jr_{vv}\right)/2\\ r_{x-RHCP} & =r_{y-LHCP}=\left(r_{uu}-jr_{vv}\right)/2 \end{array}.$$ (4)

In addition, the CP-to-LP polarization converter can be regarded as a lossless structure because the loss tangent of its two dielectric layers is only 0.0008, the magnitudes of *r*_*uu*_ and *r*_*vv*_ in the reflection matrix **R**_lin_ will both be very close to 1.0, but the phases will be different, thus, when we regard the polarization converter as a lossless structure, the following equation is established:

$$r_{vv}=r_{uu}e^{-j\Delta \varphi_{uv}},$$ (5)

wherein −180° < Δ*φ*_*uv*_ = Arg(*r*_*uu*_) − Arg(*r*_*vv*_) ≤ +180°. In this way, the CP-to-LP reflection coefficients in Eq (4) can be expressed as:

$$\begin{array}{ll} r_{y-RHCP} & =r_{x-LHCP}=r_{uu}\left(1+je^{-j\Delta \varphi_{uv}}\right)/2\\ r_{x-RHCP} & =r_{y-LHCP}=r_{uu}\left(1-je^{-j\Delta \varphi_{uv}}\right)/2 \end{array}.$$ (6)

According to Eq (6), it is known that the magnitudes of the CP-to-LP reflection coefficients can be expressed as:

$$\begin{array}{ll} \left|r_{y-RHCP}\right|=\left|r_{x-LHCP}\right| & =\left|\left(1+je^{-j\Delta \varphi_{uv}}\right)/2\right|=\sqrt{\left[1+\text{cos}\left(\Delta \varphi_{uv}-90^{{}^{\circ}}\right)\right]/2}\\ \left|r_{x-RHCP}\right|=\left|r_{y-LHCP}\right| & =\left|\left(1-je^{-j\Delta \varphi_{uv}}\right)/2\right|=\sqrt{\left[1-\text{cos}\left(\Delta \varphi_{uv}-90^{{}^{\circ}}\right)\right]/2} \end{array},$$ (7)

in addition, the following equation is established:

$$\frac{r_{y-RHCP}}{r_{x-RHCP}}=\frac{r_{x-LHCP}}{r_{y-LHCP}}=\frac{1+je^{-j\Delta \varphi_{uv}}}{1-je^{-j\Delta \varphi_{uv}}}=\frac{j\text{sin}(\Delta \varphi_{uv}-90^{{}^{\circ}})}{1-\text{cos}(\Delta \varphi_{uv}-90^{{}^{\circ}})}.$$ (8)

Eq (7) indicates that if the phase difference Δ*φ*_*uv*_ is equal to +90°, the magnitude of *r*_*y*−*RHCP*_ and *r*_*x*−*LHCP*_ will be equal to 1.0, the reflected wave under RHCP incidence will be converted to a *y*-polarized one, and the reflected wave under LHCP incidence will be a *x*-polarized one; in addition, if Δ*φ*_*uv*_ = −90°, the magnitude of *r*_*x*−*RHCP*_ and *r*_*y*−*LHCP*_ will be 1.0, the reflected waves under RHCP and LHCP incidences will be converted to *x*-polarized and *y*-polarized ones, respectively. However, if Δ*φ*_*uv*_ ≠ ±90°, the prefect CP-to-LP polarization conversion can’t be achieved, the reflected wave will be an elliptically polarized one, Eq (8) indicates that the ratio between *r*_*y*−*RHCP*_(*r*_*x*−*LHCP*_) and *r*_*x*−*RHCP*_(*r*_*y*−*LHCP*_) is a pure imaginary number, which means that the phase difference between them will be just ±90°, thus, the AR of the reflected wave can be expressed as:

AR=ry−RHCPrx−RHCP=rx−LHCPry−LHCP=sin(Δφuv−90°)1−cos(Δφuv−90°)ifry−RHCP≥rx−RHCPrx−RHCPry−RHCP=ry−LHCPrx−LHCP=1−cos(Δφuv−90°)sin(Δφuv−90°)ifry−RHCP<rx−RHCP. (9)

**Fig 2:**
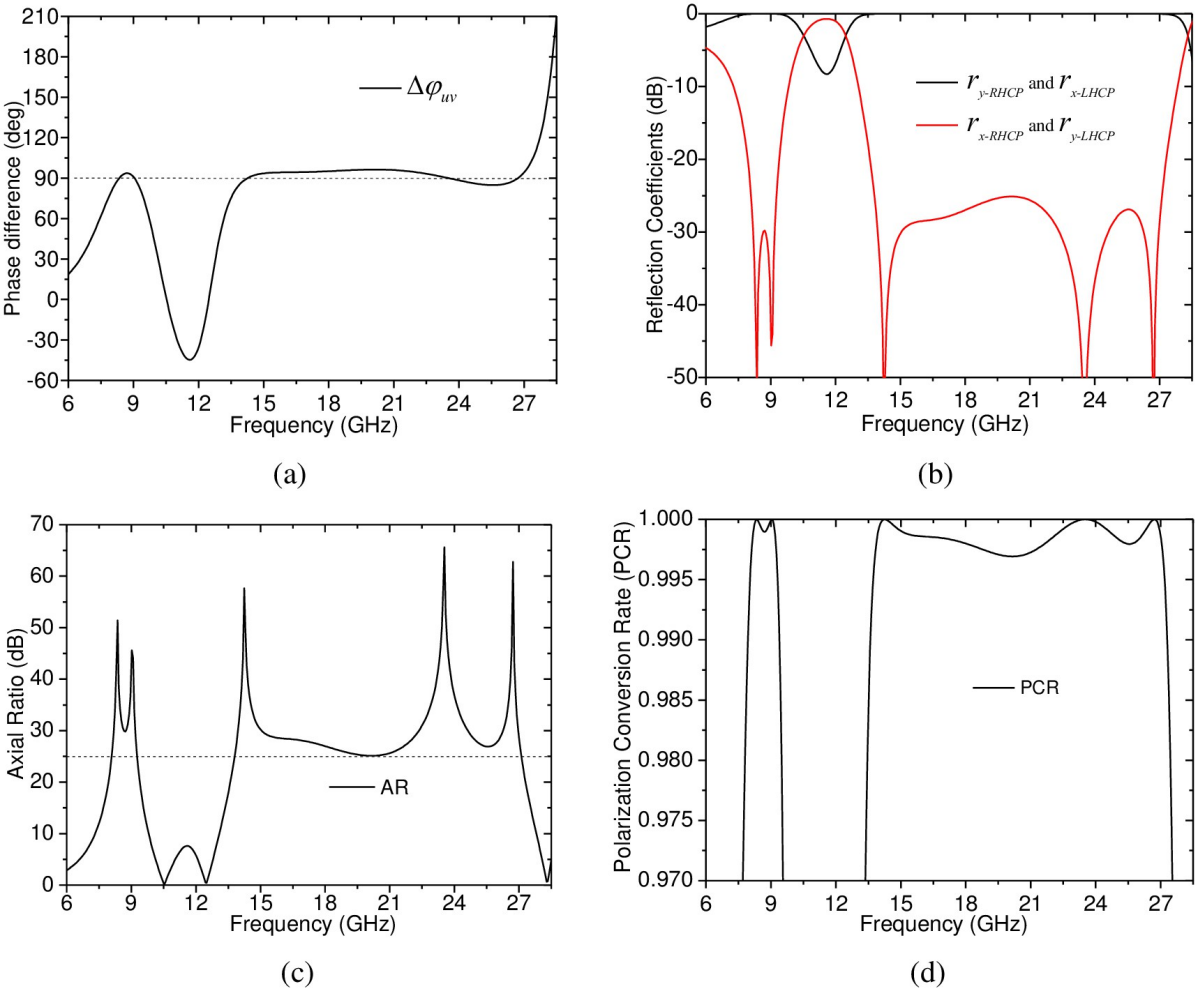
Simulation results of the proposed dual-band CP-to-LP polarization converter under *u*- and *v*-polarized incidences. (a) the phase difference Δ*φ*_*uv*_ between *r*_*uu*_ and *r*_*vv*_; (b) the predicted CP-to-LP reflection coefficients; (c) the predicted AR of the reflected wave; and (d) the predicted PCR.

To numerically investigate the performance of the proposed CP-to-LP polarization converter, we have carried out numerous numerical simulations using Ansoft HFSS. Firstly, the polarization converter was simulated under *u*- and *v*-polarized incidences, the simulation result, the phase difference Δ*φ*_*uv*_ between *r*_*uu*_ and *r*_*vv*_, is shown in Fig 2(a), it is indicated that the phase difference Δ*φ*_*uv*_ is very close to +90° in the two frequency ranges of 8–9.5 GHz and 13.5–27.5 GHz. Thus, according to the above theoretical prediction, it is known that the polarization converter can achieve CP-to-LP polarization conversion in the two separated frequency ranges, and its reflected waves under RHCP and LHCP incidences will be converted to *y*-polarized and *x*-polarized ones, respectively. To show this, according to Δ*φ*_*uv*_ in Fig 2(a), the magnitude of the CP-to-LP reflection coefficients is calculated by using Eq (7), in addition, the AR of the reflected wave is calculated by using Eq (9). The calculated results, shown in Fig 2(b) and 2(c), indicate that the magnitude of |*r*_*x*−*RHCP*_| and |*r*_*y*−*LHCP*_| is kept less than –25 dB and the AR is always larger than 25.0 dB in the two separated frequency bands of 8.08–9.27 GHz and 13.80–27.11 GHz, which shows that the proposed CP-to-LP polarization converter can achieve dual-band CP-to-LP polarization conversion in the two frequency bands. In addition, for AR=ry−RHCPrx−RHCPorrx−RHCPry−RHCP, the polarization conversion rate (PCR) of the CP-to-LP polarization conversion can be expresses as:

PCR=ry−RHCP2ry−RHCP2+rx−RHCP2orrx−RHCP2ry−RHCP2+rx−RHCP2=AR2AR2+1. (10)

Based on the CP-to-LP reflection coefficients and AR shown in Fig 2(b) and 2(c), the calculated PCR is kept larger than 99.7% in the two separated frequency bands (8.08–9.27 GHz, 13.80–27.11 GHz), as shown in Fig 2(d), it is predicted that the anticipated dual-band CP-to-LP polarization conversion can be achieved with high efficiency.

**Fig 3:**
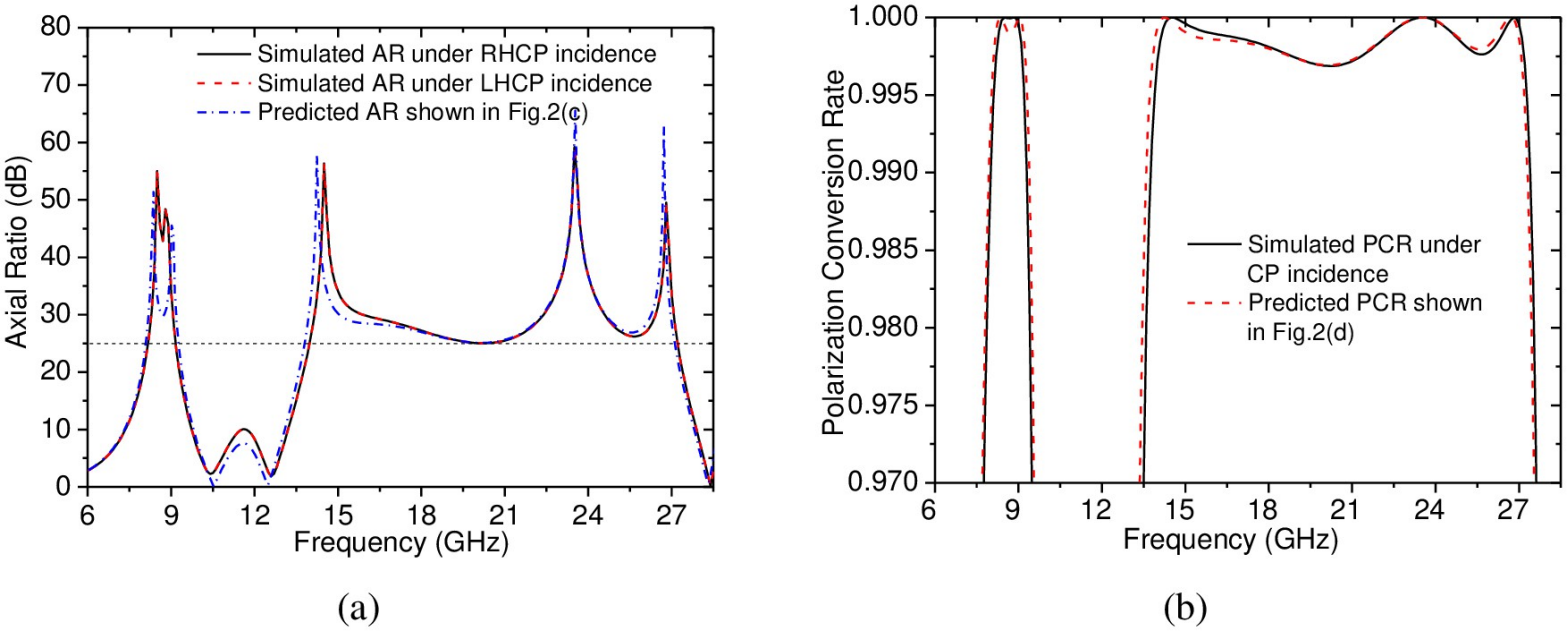
Simulation results of the proposed dual-band CP-to-LP polarization converter under CP incidence. (a) the AR of the reflected wave; (b) the PCR of the CP-to-LP polarization conversion.

Furthermore, to validate the above theoretical prediction, the proposed dual-band CP-to-LP polarization converter has been simulated directly under RHCP and LHCP incidences. The simulation was performed using the commercial software Ansoft HFSS. In the simulation, when the excitation was assumed as a CP plane incident wave, the commonly used Floquet Port could not be available, the excitation must be changed as an incident wave. In this way, in order to analyze the performance of the polarization converter in a certain frequency band, these frequencies must be swept in a discrete manner, thus, the amount of simulation calculation was increased by a little, however, the AR of the reflected wave under the CP plane-wave incidence was directly obtained through far-field report. The simulated results are presented in Fig 3(a), it is shown that the ARs of the reflected waves under the RHCP and LHCP incidences are both basically the same as the above predicted values, and they are kept larger than 25.0 dB the two separated frequency bands of 8.22–9.16 GHz and 13.98–27.21 GHz, which verify that the predicted dual-band CP-to-LP polarization conversion can be achieved by the CP-to-LP polarization converter under both RHCP and LHCP incidences. In addition, based on the ARs, the PCR of the CP-to-LP polarization conversion is calculated using Eq (10), the calculated results shown in Fig 3(b) indicate that the PCR is always larger than 99.7% in the two separated frequency bands (8.22–9.16 GHz, 13.98–27.21 GHz), which is basically agree with the predicted values shown in Fig 2(d), it is verified that the dual-band CP-to-LP polarization conversion can be achieved with high efficiency.

## 3. Experiment verification

**Fig 4:**
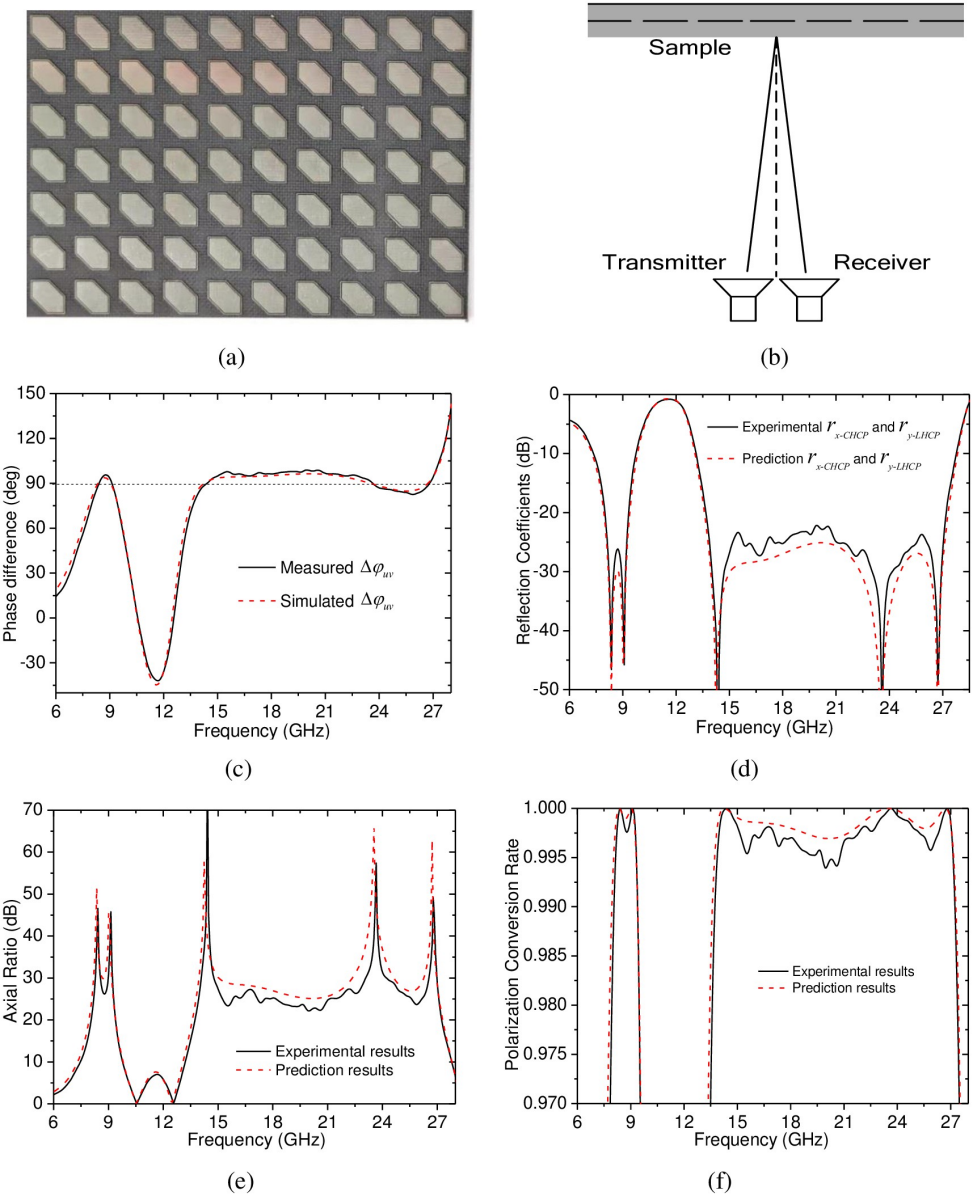
Photographs of the experimental sample (a), the schematic of the measurement setup (b), the experimental results: (c) the phase difference Δ*φ*_*uv*_; (d) the AR of the reflected wave under CP incidences; (e) the AR of the reflected wave under CP incidences; (f) the PCR of the CP-to-LP polarization conversion.

In order to experimentally validate the performance of the proposed CP-to-LP polarization converter, an experimental sample was fabricated and measured. The grounded dielectric substrate of the experimental sample is shown in Fig 4(a), which consists of 35×35 unit cells with an area of 280mm×280mm. When the grounded dielectric substrate was hot pressed together with the top dielectric layer, the sample was constructed, it was measured in a microwave-darkroom by using an Agilent E8363B network analyzer together with a pair of identical standard-gain horn antennas, the schematic illustration of the measurement setup is shown in Fig 4(b), in which the transmitter and receiver were used to transmit the incident wave and receive the reflected wave, respectively, the sample was irradiated, and the distance between the sample and the transmitter and receiver was 1.0 m; in addition, to carry out the measurement under normal incidence, the separation angle between the orientations of the transmitter and receiver should ideally be close to 0°, it was set to be 6° for the finite sizes of the experimental sample. The sample has only been measured under *u*- and *v*-polarized incidences because no ultra-wideband CP antenna exists in common microwave experimental equipment. The measured result, the phase difference Δ*φ*_*uv*_ between *r*_*uu*_ and *r*_*vv*_, is shown in Fig 4(c), it is indicated that the measured Δ*φ*_*uv*_ is close to +90° in the two frequency ranges of 8–9.5 GHz and 13.5–27.5 GHz, which reasonably agrees with the above simulated results in Fig 2(b). Thus, based on the measured Δ*φ*_*uv*_, the calculated magnitudes of the CP-to-LP reflection coefficients, together with the calculated AR of the reflected wave, are basically the same with the above predicted values, as shown in Fig 4(d) and 4(e), in addition, based on the calculated AR, the calculated PCR, shown in Fig 4(f), is also agree well with the predicted results, it is furtherly proved that the CP-to-LP polarization converter can achieve high-efficiency dual-band CP-to-LP polarization conversion under both RHCP and LHCP incidences.

## 4. Conclusions

In this paper, a high-efficiency and dual-band CP-to-LP polarization converter was proposed based on an anisotropic metasurface. Because the proposed polarization converter is an orthotropic anisotropic structure with a pair of mutually perpendicular symmetric axes *u* and *v*, and the reflection phase difference Δ*φ*_*uv*_ under *u*- and *v*-polarized incidences is very close to +90° in two separated frequency ranges, the polarization converter can convert RHCP wave into *y*-polarized one and convert LHCP wave into *x*-polarized one with a PCR greater than 99.7% in the two separated frequency bands of 8.08–9.27 GHz and 13.80–27.11 GHz. The performance of the CP-to-LP polarization converter has been verified by both simulations and experiment. In summary, the proposed polarization converter can achieve CP-to-LP polarization conversion, moreover, it is not only dual band, but also highly efficient, so it is of great application value to modulate the polarization of various EM waves.

## Supporting information

S1 Dataset (RAR)
